# Dynamic Entropy of Two-Dimensional Active Brownian Systems in Colloidal Plasmas

**DOI:** 10.3390/molecules27051614

**Published:** 2022-02-28

**Authors:** Xeniya G. Koss, Evgenii A. Kononov, Irina I. Lisina, Mikhail M. Vasiliev, Oleg F. Petrov

**Affiliations:** 1 Joint Institute for High Temperatures, Russian Academy of Sciences, 125412 Moscow, Russia; gadvin@yandex.ru (E.A.K.); irina.lisina@mail.ru (I.I.L.); mixxy@mail.ru (M.M.V.); ofpetrov@ihed.ras.ru (O.F.P.); 2 Moscow Institute of Physics and Technology, 141700 Dolgoprudny, Russia; 3 Center for Astrophysics, Space Physics and Engineering Research (CASPER), Baylor University, Waco, TX 76798, USA

**Keywords:** colloidal plasmas, Brownian motion, active Brownian particles, MFPT dynamic entropy, fractal dimension

## Abstract

We analyze the experimental data on the motion of active Brownian micrograins in RF discharge plasmas. In the experiments, two types of microparticles were used: first—plastic grains fully covered with metal, and second—Janus particles with a thin metal cap. We have tracked the trajectories of the separate grains and plotted the pair correlation functions of the observed structures. To examine the motion of the grains, we studied the dependencies of the MFPT dynamic entropy on the coarsening parameter, the fractal dimension of the system on its mean kinetic temperature, and the mean localization area of the grain on its mean kinetic temperature. Based on the obtained results, we conclude that the character of motion of our active Brownian systems changes as the power of an illuminating laser (and, therefore, the mean kinetic temperature of the grains) increases. Janus particles change their trajectories from more chaotic to spiral-like ones; in the case of fully covered particles, we observe the dynamical phase transition from the more ordered structure to the less ordered one.

## 1. Introduction

In the last ten years, the problems considering the dynamics of so-called active Brownian, or self-propelled, particles, have become more and more essential. These particles possess the unique property to convert external energy into the kinetic energy of their motion [1,2,3]. The nature of these particles may be various: colloid grains in buffer media [4,5], protozoa and bacteria [6,7], chemically activated particles [8,9,10] and, even, mechanical objects [11,12,13,14]. Active Brownian particles and the structures made of them, thanks to their ability to transform energy, have some exceptional properties and features that are interesting both for the fundamental science and for the practical tasks—in biology, medicine, and engineering [2,15,16]. So, for example, one can observe dynamical phase transitions and phase separation in the structures of active Brownian particles that have no analogues in the passive Brownian systems [17,18]. One of the remarkable examples of such an outstanding difference is the motility-induced phase separation (MIPS), which is often observed in the systems of self-propelled particles [2,19,20].

However, the main feature of active Brownian systems is that they are always out of the equilibrium, since that presents an external energy flow. Methods of classical thermodynamics cannot be used to study these systems and processes in them, as this is essentially a non-equilibrium case. Nevertheless, the question is—what happens with these systems as they evolve? How can we describe their condition?

The cooperative behavior of non-equilibrium open systems and the processes of ordering in them (which have the nature of phase transitions) are investigated within the framework of the physics of dissipative systems developed by Prigogin [21], or the synergetics introduced by Haken [22]. The entropy and other instruments used within these concepts are universal, and they are successfully applied in various fields of knowledge [23]. In this work we use the approach presented in [24,25,26,27]—the calculation of the mean first-passage time (MFPT) dynamic entropy. With this approach, one can describe the motion of each separate (active or passive) Brownian particle with the help of several parameters (such as the fractal dimension of its trajectory and the size of the localization area), comparing the different regimes of motion with each other.

A convenient object for studying active Brownian particles is laboratory colloidal plasma. This is the system of micron-sized charged particles located in the gas discharge. It is the bright example of the system far from equilibrium: it exists only when the gas discharge is maintained. Thanks to the surface properties of the micrograins, their passive Brownian motion can be activated, i.e., becoming active Brownian. For example, if the surface of the grain is fully or partly covered with metal, the external laser radiation heats it up. The thermophoretic force arising from the interaction of the neutrals of the buffer gas with the surface of a grain adds the directed component into its chaotic passive Brownian motion. Small systems of the particles fully covered in metal are considered in [25,27]; the motion of an isolated Janus particle in RF discharge is described in [28,29]. The resulting kinetic temperature of grains in plasmas multiply exceeds the temperature of its environment (the temperature of a buffer gas is ~0.03 eV, and the characteristic temperature of grains is about 100 eV). According to [2], it is the characteristic feature of active matter.

One of the peculiarities of our experiment is that we consider the extended system of active particles, not a single grain. Moreover, the viscosity of the environment of active particles (buffer gas) is low, i.e., we deal with an underdamped motion.

Active Brownian motion can be classified as overdamped and underdamped, depending on the parameters of the medium, the particle and corresponding characteristic relaxation times) [3,30]. In the case of overdamped motion, which is studied in most experimental and theoretical works on active systems [2], inertia does not affect the motion of the particle. However, in recent years, more and more groups consider larger self-propelled particles or motion in low-density environment (gas instead of liquid). In this case, the corresponding Reynolds number for the particle motion is higher, and one should take into account inertial effects. The character of motion in these systems is different from overdamped case; the inertia-dominated particles are “microflyers” rather than “microswimmers”. [3]. As examples of these “flying” active particles, one can mention granulars self-propelling on a vibrating plate or having a vibrational motor [11,13], minirobots [12], insects [31] and micrograins in plasmas, considered in this work. In these systems, motility-induced phase separation (MIPS) occurs substantially less often than in overdamped ones [3]. In addition, overall, the dynamics of active Brownian systems in gas-discharge plasmas essentially differ from the behavior of active colloids in liquid buffer media, as the viscosity of buffer gas is vanishingly small. We should also note that active systems in plasma consisting of colloids interacting via long-ranged electrostatic potential [32,33,34] represent a system that lacks both experimental implementation and theoretical description. Numerical simulations of such underdamped systems and theoretical predictions for their parameters are presented in [30].

In the present work, we study the underdamped motion of two kinds of active Brownian particles in plasma: laser-activated grains (fully covered with metal) and Janus particles (half-covered with metal). In both experiments, active grains form a large structure (several hundreds of particles), unlike the previous experimental works on active systems in colloidal plasmas (only isolated active particles [28,29] or small systems [25,27] were considered before). To analyze the motion of grains, we used the MFPT dynamic entropy approach, that is a convenient instrument showing the degree of chaotization of the system [24,25,26,27].

## 2. Results

In this article, we consider two series of experiments on formation of extensive structures (consisting of several hundreds) of micron-sized particles in the near-electrode area of RF discharge. When the grains fall into plasmas, they acquire an electrical charge and levitate under the influence of the external electrical field and the gravitational force. As a result of interaction with one another and the electrical trap (the metal ring), the grains form structures of various degrees of order. Note, also, that the micrograins in our experimental conditions form a single layer, i.e., a quasi-two-dimensional structure. The motion of the grains inside the layer is composed of the classic Brownian (due to the collisions with the atoms of buffer gas) and the active (induced by the external laser radiation).

In the first series of experiments, we used spherical melamin-formaldehyde particles with a thin solid copper covering. The diameter of the particles was 10.0 μm. The diameter of the trap ring was 60 mm. For the visualization of the obtained structure and influencing it, we used the flat wide laser beam (10 × 100 mm). The power of the laser was varied from 0.019 W to 0.88 W.

In the second series of experiments, we used melamin-formaldehyde spherical particles having thin metal caps (so-called Janus particles). The technique of production of these grains is detailed in [29,35]. The diameter of particles was 10.2 μm. The diameter of the trap ring was 60 mm. As well as in the experiments with covered particles, the obtained structure was illuminated by flat laser beam. Its power varied from 0.07 W to 6.6 W.

The action of laser radiation on the structure was continuously registered, with the help of video camera with a frame rate 400 fps and a resolution 1440 × 1440 pixels. From the captured video recordings, we were able to restore the coordinates of the particles in each moment of time, their trajectories and velocities.

**Figure 1:**
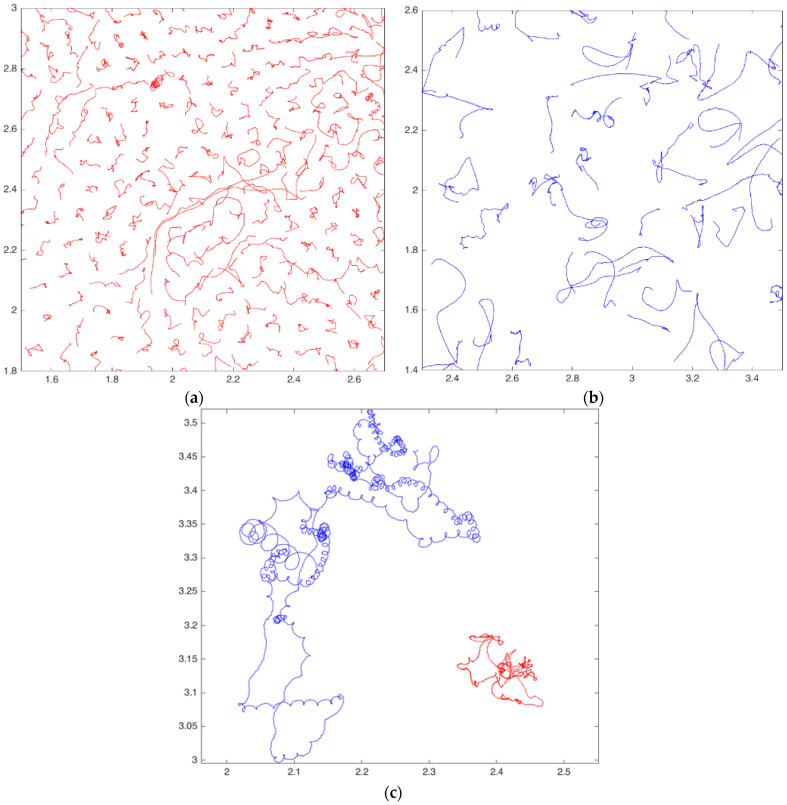
Trajectories of: (**a**) the fragment of the system of metal-covered grains for 1 s, *W* = 0.14 W; (**b**) the fragment of the system of Janus particles for 1 s, W = 0.13 W; (**c**) one of the fully covered grains (red curve) and one of the Janus particles (blue curve) from the systems shown in Figure 1a,b for the whole observation time. View from the top. The distance along the coordinate axes is expressed in centimeters.

Figure 1a–c shows the trajectories of structures of covered and Janus particles. To demonstrate the structure as a whole (Figure 1a,b), we have chosen the “coldest” examples: the laser power in experiments with covered and Janus particles was rather low (0.14 W and 0.13 W, correspondingly). One can see that the Janus structure, unlike the structure consisting of the covered particles, does not have any order, and Janus particles have considerably longer trajectories than covered ones for the same timespan. Note that the trajectory of Janus particle has characteristic parts corresponding to the motion of active chiral particles (so-called “circle swimmers”) [28,36,37]—circles, spirals and epitrochoids. In contrast to it, the path of the covered grain represents a chaotic curve. The character of the track of a grain obviously depends on the peculiarities of its form and coating, as well as on the number of grains in the neighborhood. Apparently, the chirality of our Janus particles is caused by the asymmetry of the metal coating.

**Figure 2:**
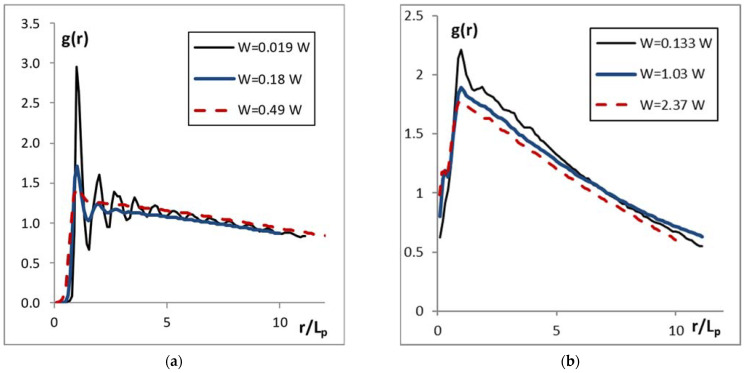
Pair correlation functions of (**a**) the system of metal-covered grains and (**b**) the system of Janus particles for various powers of illuminating laser W (see the legend). The values of the mean interparticle distance *L*_p_ for each considered system were as follows. Panel (**a**): W = 0.019 W, *L*_p_ = 0.09 cm; W = 0.18 W, *L*_p_ = 0.09 cm; W = 0.49 W, *L*_p_ = 0.088 cm. Panel (**b**): W = 0.133 W, *L*_p_ = 0.18 cm; W = 1.03 W, *L*_p_ = 0.18 cm; W = 2.37 W, *L*_p_ = 0.2 cm.

For each of the observed structures, we plotted the pair correlation functions (PCFs) and found the mean interparticle distances *L*_p_. Examples of the pair correlation functions for the systems with various degrees of ordering are presented in Figure 2.

The choice of ranges of W to display in panels (a) and (b) of Figure 2 was determined by the specificity of the experiment. We have set the laser power so that the illuminated dusty system was still stable. Fully covered grains (panel (a)) acquired relatively high kinetic temperature even when the laser power was small, and for W ≥ 1 W the system was already destroyed. For Janus particles (panel (b)), the comparable kinetic temperatures were reached with the laser power much higher than for the fully covered particles. The explanation probably is in the difference of metal-covered areas of grains.

For the system of covered particles, one can distinguish the curves characteristic of the two-dimensional crystals and liquid-like structures. As this structure heats up, it passes through several phase states [38], which are shown in panel (a): here one can see the PCFs characteristic of the strongly coupled system (W = 0.019 W), the weakly coupled system (W = 0.49 W) and the system with an intermediate degree of correlation (W = 0.18 W).

The PCFs of Janus systems differ considerably. Despite that the magnitude of the first peak here also decreases with the increase in laser power W, the oscillations in PCFs are absent even for the lowest value of W (0.13 W), and it decreases quickly with the distance, which points to the absence of correlation between Janus particles. The three values of W in panel (b) are chosen for illustration purpose, as the minimal, maximal and average laser power.

We have found the mean kinetic temperature of dust grains T for each experiment. We have observed essentially nonlinear direct dependence T(W). The variation of the kinetic energy of the grains is mainly caused by the action of the photophoretic force [27,39,40]:

F_ph_ = (πa^3^∙p∙*I*)/(6 k_th_∙T_buf_), (1)

where a is the radius of a particle, p is the pressure of a buffer gas, T_buf_ is the temperature of a buffer gas, *I* is the intensity of the laser radiation and k_th_ is the thermal conductivity of a particle. Assuming that all these parameters (except *I*) remain constant during the experiment, one can come to a conclusion that the nonlinearity of T(W) is induced by the closest neighbours of the colloidal particle. So, the above-mentioned relationship for F_ph_ serves only as orientation point that explains the nature of activity of the considered particles. The quantitative explanation of the dependence T(W) should be the topic of a separate research.

**Figure 3:**
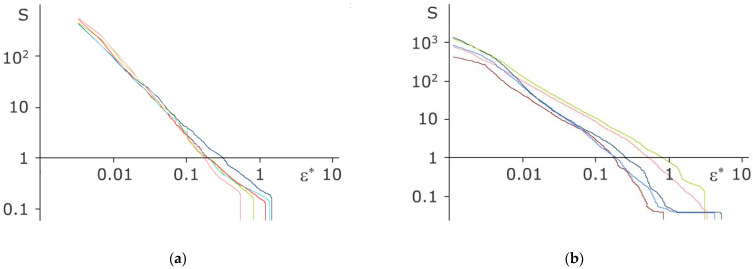
The dependencies of MFPT dynamic entropy on the normalized coarsening parameter for five random (**a**) metal-covered grains under the influence of laser power W = 0.019 W; (**b**) Janus particles under the influence of laser power W = 0.13 W.

For all the considered systems of covered and Janus particles, we have calculated the dependencies of MFPT dynamic entropy on the normalized coarsening parameter ε^∗^ = ε/*L*_p_, where *L*_p_ is the mean interparticle distance. Some examples of these dependencies for random covered and Janus particles are shown in Figure 3.

The beginning of the curves S(ε^∗^) (data for small ε^∗^) corresponds to the ballistic regime of particle motion. All particles in the structure in this regime move almost identically, and the logarithmic derivative d(lg(S(ε^∗^)))/d(lg ε^∗^) is equal to −1 with good accuracy. After the range corresponding to the ballistic mode, the magnitude of the derivative for Brownian particles should grow [26,27]. For large values of ε^∗^, the magnitude of the derivative d(lg(S(ε^∗^)))/d(lg ε^∗^) becomes constant; its value shows the fractal dimension of trajectory of a particle (and the value Δ_f_ = < d(lg(S(ε^∗^)))/d(lg ε^∗^) > averaged over the ensemble of particles characterizes the fractal dimension of a system as a whole). However, we can see that for fully covered particles the slope changes very weakly for all ε^∗^ considered. In addition, in fact, it results in low fractal dimension (Δ_f_ ~ 1.3 the laser power W = 0.019 W). For Janus particles, the change of slope after ballistic regime is more noticeable (around ε^∗^ ~ 0.008), but it is still not large. It results in higher fractal dimension (Δ_f_ ~ 1.6 for the laser power W = 0.13 W).

## 3. Discussion

**Figure 4:**
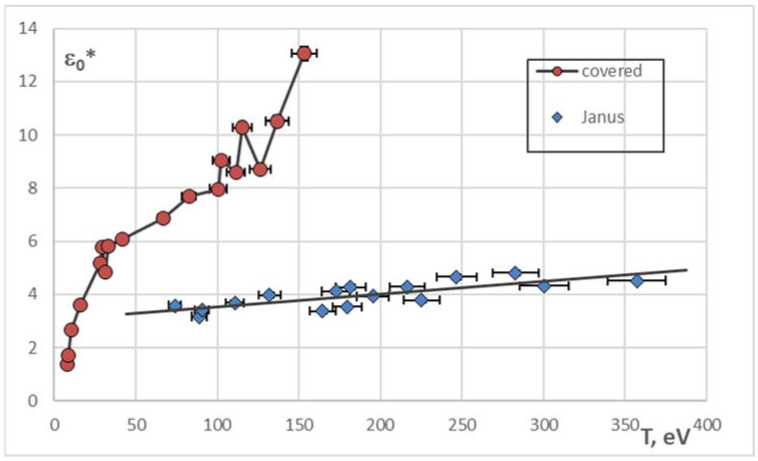
The dependence of mean localization area of ○—metal-covered and ◊—Janus particles on their mean kinetic temperature.

For all the conducted experiments, we have calculated the sizes of localization areas of colloid grains, i.e., the radii of the circles that contain their trajectories. This parameter can be easily obtained from the MFPT dynamic entropy: it is the coarsening parameter ε_01_*, such that for every ε* > ε_01_* holds S(ε*) = 0 [26,27]. As the number of particles in the system under consideration is rather large (several hundreds), we calculated the average value ε_0_* = <ε_01_*> as a characteristic quantity. The dependence of ε_0_* on the kinetic temperature is shown in Figure 4.

The size of the localization area for all the experiments is higher than 1. It contradicts the results of numerical simulation of the extensive systems of passive Brownian particles [26] and the experiments with the clusters of active Brownian particles [27], where in the crystalline state (or crystalline-like, in case of clusters) ε_0_* < 1, i.e., the areas where the separate particles move are isolated from one another. The overlapping of the localization areas of the particles, even for the “coldest” structures in our case, can be caused by the higher activity of the grains than in [27] (due to the features of the surface or the laser power), as well as by the influence of the trap.

The plot of ε_0_*(T) for the metal-covered particles has the inflection point T_1_ ~ 30 eV. It corresponds to the phase transition “dust crystal—dust liquid”. Moreover, the sharp increase in the localization area is obvious for T > T_2_ ~ 120 eV.

For Janus particles, we observed the monotonous increasing of the localization area for all the temperature interval explored (see the trendline in Figure 4).

A slope of the dependence S(ε*), i.e., the derivative d(lg(S(ε^∗^)))/d(lg ε^∗^), is also informative. For small values of ε* (in the ballistic regime of motion), it is close to -1; for distances approaching the limiting circle (ε*→ε_01_*), it tends to the value corresponding to the fractal dimension of the grain’s trajectory: the path of the passive, as well as the active, Brownian particle is a fractal, i.e., a self-similar object [41]. Unlike the spatial dimension, the fractal dimension does not have to be an integer. For example, the spatial dimension of a flat curve is 1, but the fractal dimension of a trajectory of the passive Brownian particle on a plane is 2. So, the fractal dimension of a pattern shows its space-filling capacity.

**Figure 5:**
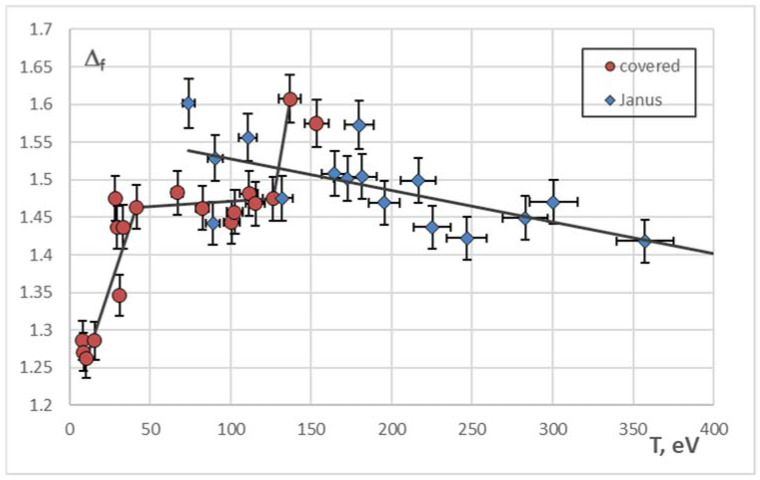
The dependence of mean fractal dimension of the trajectories for ○—metal-covered and ◊—particles on their mean kinetic temperature.

The constant Δ_f_ = −< d(lg(S(ε^∗^)))/d(lg ε^∗^) > averaged over the ensemble of particles shows the fractal nature of the system of active grains [24,41]. This approach was successfully used to study the small structures of active Brownian particles [27]; its validity was proved by the comparison with the results of numerical simulation of extensive Brownian systems [26]. The dependencies Δ_f_(T) for the experiments with metal-covered and Janus particles are shown in Figure 5.

The curve plotting the dependence Δ_f_(T) for metal-covered particles has two special points T_1_ and T_2_, corresponding to the special points on the plot ε_0_*(T) (Figure 4). The point T_1_ ~ 30 eV seems to correspond to the dynamic phase transition from the more ordered state to the less ordered (“crystal-liquid” transition). On the figures plotting the trajectories of all the particles in the structure, after passing this point the trajectories of the separate particles become indistinguishable. Further, for the range of temperatures 30–100 eV the fractal dimension of trajectories changes slightly and is close to 1.5—the value of the fractal dimension of trajectories of active Brownian clusters at high temperatures [27]. For T > T_2_ ~ 120 eV Δ_f_ of metal-covered particles experiences a jump. This jump demonstrates the transition to the state when the particle begins to “feel” the trap, and its trajectory covers the plane denser. Nevertheless, the question about the kinetics of metal-covered particles at these temperatures is still open and needs to be studied closely.

The fractal dimension of trajectories of Janus particles decreases as the structure heats (see the trendline in Figure 5), which speaks for the increasing of amount of the spiral and circular segments in them. As the temperature rises, the active component of the Brownian motion begins to prevail the chaotic (passive) component.

## 4. Materials and Methods

We have experimentally obtained the extensive quasi-two-dimensional systems consisting of the two types of grains. The grains of the first type have 10 μm in diameter and are made of plastic, completely covered with a thin layer of copper. These particles are produced by microParticles GmbH [42]. The second type of grains, Janus particles, are made of plastic and half-coated with metal. The plastic basis of these particles was also purchased from microParticles GmbH [42], and the metal caps were applied by ourselves. A detailed methodology for making such Janus particles is presented in [35].

The experiments with both types of grains were carried out in RF-discharge plasma. The discharge chamber had optical windows for laser radiation input and observations. After pumping to vacuum, the gas-discharge chamber was filled with argon to a pressure of 5–6 Pa. A voltage of 300 V with a frequency of 13.56 MHz was applied to the horizontally set electrodes, igniting the discharge. On the lower electrode, we have placed a metal ring (60 mm in diameter) that created a potential trap for the particles. A detailed scheme and description of the experimental setup can be found in [29]. In this configuration of setup, the micrograins formed an extensive quasi-two-dimensional structure.

The considered systems of active Brownian particles are essentially out of equilibrium: their dynamics is defined by the input of an external energy and its transformation into the kinetic energy of the particle motion. So, the techniques for the analysis of the state of active Brownian systems should not be based on thermodynamic isolation of a system under study.

The dynamic entropy, proposed in [25,26,27] as an approach to describe the kinetics of active Brownian systems, satisfies this condition. It is known that the maximal Lyapunov exponent has its extremum in the point of “liquid-solid” phase transition [43]. The position of this extremum corresponds to the maximum of the Kolmogorov-Sinai entropy $\underset{i}{\sum }\lambda_{i}=h_{\mathrm{KS}}\;$ that is the sum of the Lyapunov exponents. However, we cannot apply Kolmogorov-Sinai entropy “as is” to analyze the Brownian systems, as the trajectories of Brownian particles are nondifferentiable, and *h*_KS_ diverges [24,44]. That is why, in case of such stochastic systems, it is more convenient to use the dynamic entropy *h*(ε) dependent on the partitioning parameter ε [44]. In the present work, we use an approach called the “mean first-passage time dynamic entropy” [24,44,45].

Assuming that the spatial scale is not too small [44], one can approximately calculate the dynamic entropy in the following way. One should draw a sphere of radius ε in the moment of time t = 0, so that its center coincides with the location of the particle. Then, find the moment of time τ, when the trajectory of the particle first crosses the threshold ε. Averaging this mean first-passage time (MFPT) τ(ε) over all the particles of the system, we find the MFPT entropy S(ε) [22]:

S(ε) ≡ 1/τ(ε),(2)

where $\tau \left(\varepsilon \right)=\int_{0}^{\infty }P_{\varepsilon }\left(t\right)\mathrm{tdt}$, and P_ε_(t) is the probability that the particle reaches the boundary ε in the moment of time between t and t + dt.

The further a particle goes from its initial position, the smaller S(ε) is. Eventually, when the radius ε of the drawn sphere becomes large enough, the whole particle trajectory is contained in it. This situation corresponds to the point ε = ε_0_, when S(ε) abruptly goes to zero (see Figure 3 for illustration).

If we consider the distribution of particle displacements to be Gaussian, then their mean-square displacements will follow the scaling law [24]

(3) $$\langle r^{2}\left(t\right)\rangle \propto t^{2\nu },\;$$

and the first-passage time

(4) $$\tau \left(\varepsilon \right)\propto \varepsilon^{1/\nu }$$

Here, ν is the constant depending on the characteristics of the system and on the time scale of observation. For example, ν = 1 is observed in the short time inertial regime. For very long times, if the particle displacement distribution is Gaussian (e.g., for the passive Brownian motion), then ν = ½.

Consider Δ(ε) = −d(lg(S(ε)))/d(lg ε)—a logarithmic derivative of S(ε). Then, at large ε

(5) $$\Delta \left(\varepsilon \right)\to \Delta_{f}=\frac{1}{\nu^{’}}$$

which corresponds to the fractal dimension of the particle trajectory.

For the systems with motion types different from passive Brownian, the value of Δ(ε) at large ε may differ from 2. The values of 1 < Δ_f_ < 2 correspond to the persistent fractal Brownian motion, the values of Δ_f_ > 2—to the antipersistent fractal Brownian motion [41].

Note that S(ε), as well as its logarithmic derivative, is discontinuous at ε = ε_0_. We take Δ_f_ = −< d(lg(S(ε^*^)))/d(lg ε^*^) > as the mean value of left-hand derivatives at ε^*^ → ε_01_* for each S(ε^*^).

Analyzing the dependencies of MFPT dynamic entropy and its derivative on the coarsening parameter ε, we obtain the localization area and mean fractal dimension of the grain trajectories that allow us to describe the degree of chaotization of the system [25,26].

## 5. Conclusions

We have analyzed the experimental data on the motion of active Brownian micrograins in RF-discharge plasmas. Two types of microparticles were considered: (1) plastic grains fully covered with metal; and (2) Janus particles with a thin metal cap. The trajectories of separate grains were obtained, pair correlation functions of the structures were plotted. We have studied the dependencies of the MFPT dynamic entropy on the coarsening parameter, the fractal dimension of the system on its mean kinetic temperature, and the mean localization area of the grain on its mean kinetic temperature. Based on the obtained results, we conclude that the character of motion of our active Brownian systems changes as the power of the illuminating laser (and, therefore, the mean kinetic temperature of the grains) increases. We have revealed two critical points in the dependency Δ_f_(T) for metal-covered particles; the first one, T_1_ ~ 30 eV, corresponds to the dynamical phase transition from the more ordered to the less ordered state (“crystal-liquid”). The second one, T_2_ ~ 120 eV, gives evidence that the path of a grain becomes comparable to the size of the electromagnetic trap.

For the Janus particles, we have observed the monotonous growth of the mean localization area and the decrease in the mean fractal dimension of their trajectories with increasing kinetic temperature T. That is the evidence that the motion of Janus particles becomes less chaotic and more directed as the system warms up.

Systems of active Brownian particles represent an essential and rapidly developing branch of modern science involving physics, chemistry, biology and medicine. The approach to their study presented in this paper allows one to easily observe and evaluate the transition of such systems (or their parts) from the ordered to chaotic state.

## Data Availability

Not applicable.

## Sample Availability

Not available.
